# IPA-3 Inhibits the Growth of Liver Cancer Cells By Suppressing PAK1 and NF-κB Activation

**DOI:** 10.1371/journal.pone.0068843

**Published:** 2013-07-19

**Authors:** Leo Lap-Yan Wong, Ian Pak-Yan Lam, Tracy Yuk-Nar Wong, Wai-Lung Lai, Heong-Fai Liu, Lam-Lung Yeung, Yick-Pang Ching

**Affiliations:** 1 Department of Anatomy, Li Ka Shing Faculty of Medicine, The University of Hong Kong, Hong Kong, China; 2 Center for Cancer Research, Li Ka Shing Faculty of Medicine, The University of Hong Kong, Hong Kong, China; 3 Department of Chemistry, Hong Kong University of Science and Technology, Hong Kong, China; 4 State Key Laboratory of Liver Research, Li Ka Shing Faculty of Medicine, The University of Hong Kong, Hong Kong, China; 5 State Key Laboratory of Brain and Cognitive Science, Li Ka Shing Faculty of Medicine, The University of Hong Kong, Hong Kong, China; University of Alabama at Birmingham, United States of America

## Abstract

Hepatocellular carcinoma (HCC) is one of the major malignancies worldwide and is associated with poor prognosis due to the high incidences of metastasis and tumor recurrence. Our previous study showed that overexpression of p21-activated protein kinase 1 (PAK1) is frequently observed in HCC and is associated with a more aggressive tumor behavior, suggesting that PAK1 is a potential therapeutic target in HCC. In the current study, an allosteric small molecule PAK1 inhibitor, IPA-3, was evaluated for the potential in suppressing hepatocarcinogenesis. Consistent with other reports, inhibition of PAK1 activity was observed in several human HCC cell lines treated with various dosages of IPA-3. Using cell proliferation, colony formation and BrdU incorporation assays, we demonstrated that IPA-3 treatment significantly inhibited the growth of HCC cells. The mechanisms through which IPA-3 treatment suppresses HCC cell growth are enhancement of apoptosis and blockage of activation of NF-κB. Furthermore, our data suggested that IPA-3 not only inhibits the HCC cell growth, but also suppresses the metastatic potential of HCC cells. Nude mouse xenograft assay demonstrated that IPA-3 treatment significantly reduced the tumor growth rate and decreased tumor volume, indicating that IPA-3 can suppress the *in vivo* tumor growth of HCC cells. Taken together, our demonstration of the potential preclinical efficacy of IPA-3 in HCC provides the rationale for cancer therapy.

## Introduction

As the sixth most common malignant tumor and the third leading cause of cancer mortality worldwide, hepatocellular carcinoma (HCC) is responsible for more than a million deaths annually [1]. HCC is associated with poor prognosis due to high incidences of tumor recurrence and metastasis [2]. Liver resection is one of the major therapies at present but remains unsatisfactory because of the high recurrence rates [3]. Therefore, the development of novel treatment regimens for HCC is required.

Overexpression of p21-activated kinase 1 (PAK1) is frequent in HCC [4]. It is a downstream effector of the small Rho GTPase, including Rac1 and Cdc42, which regulates diverse cellular processes, including cell cycle progression, cell motility, cell polarity and apoptosis [5]. Activated Rho GTPase binds to PAKs on the Cdc42/Rac interactive binding (CRIB) domain, causing the relief of autoinhibitory domain (AID), subsequent autophosphorylation of the catalytic domain and kinase activation [6]. Among the multiple autophosphorylation sites, threonine-423 (T423) is particularly important for counteracting autoinhibition and maintaining the complete activated state [7].

IPA-3 (2,2′- dihydroxy-1,1′-dinaphthyldisuifide) is a highly selective, non-ATP-competitive allosteric inhibitor of PAK1 whose hyperactivity has been shown to be closely related with tumorigenesis [8]. Previous studies demonstrated that IPA-3 prevented Cdc42-induced PAK1 autophosphorylation on T423 and significantly inhibited PAK1 catalytic activity [8], [9]. The inhibitory action of IPA-3 is achieved in part by binding covalently to the regulatory domain of PAK1 which in turn prevented the physical interaction with Cdc42 or other GTPase activators [9]. IPA-3-targeting regulatory domain is less conserved within kinases, thus confers a remarkably high selectivity to this inhibitor [8]. *In vitro* studies showed that IPA-3 treatment led to similar results as siRNA silencing of PAK1, in which IPA-3 specifically blocked the membrane transport of WAVE2 and lamellipodia formation in human breast cancer cells [10], and inhibited the endocytic uptake of human adenovirus serotype 35 in various cell lines [11]. However, the effect of IPA-3 in the therapeutic treatment of human HCC is still poorly understood. In this study, we aimed to investigate the potential of IPA-3 in suppressing the proliferation and metastasis of human HCC cells through a series of *in vitro* and *in vivo* experiments. We showed that treatment of IPA-3 had a significant impact on the apoptosis, proliferation and motility of HCC cells. Furthermore, IPA-3 was able to suppress the *in vivo* tumor growth in nude mouse xenografts. Therefore, our data provides supportive evidences for the potential application of IPA-3 in managing tumorigenesis and metastasis of HCC.

## Materials and Methods

### Chemicals

2,2′-dihydroxy-1,1′-dinaphthyldisuifide (IPA-3) was synthesized and provided by Dr. L.L. Yeung in Hong Kong University of Science and Technology. The structure of IPA-3 was confirmed by mass spectrometry analysis. A stock solution of IPA-3 (100 mM) was freshly prepared in DMSO. Other chemicals unless specifically stated were from Sigma-Aldrich at the highest quality.

### Cell Culture

Human HCC cells H2M, H2P and the human non-tumorigenic, immortalized liver line MIHA were from Dr. X.Y. Guan, Department of Clinical Oncology, University of Hong Kong, Pokfulam, Hong Kong [12]. MHCC97L and MHCC97H cells were from Liver Cancer Institute, Fudan University, Shanghai, China [13]. HepG2 and Hep3B cells were purchased from American Type Culture Collection (ATCC). SMMC-7721 and Bel-7402 were gifts from Shanghai Institute of Biochemistry and Cell Biology, Chinese Academy of Sciences. All cells were grown in Dulbecco’s modified Eagle minimal essential medium (DMEM) with high glucose supplemented with 10% heat-inactivated fetal bovine serum (FBS), 1 mM sodium pyruvate and 100 U penicillin/streptomycin, at 37°C in humidified 5% CO_2_ incubator.

### MTT Assay

Four thousand H2M cells per well were seeded in 96-well plates and incubated in normal condition for 24 hours. Cells were treated with different concentrations of IPA-3 for 1, and 2 days. Cells were treated with 100 µl of 5 mg/ml of (3-(4,5-Dimethylthiazol-2-yl)-2,5-diphenyltetrazolium bromide (MTT) (Invitrogen) solution for 4 hours at 37°C until crystals were formed. MTT solution was removed from each well and 100 µl of DMSO was added to each well to dissolve the crystals. Color intensity was measured by Microplate Reader (Bio-Rad) at 570 nm. Each experiment consisted of four replications and at least three independent experiments were carried out.

### Cell Proliferation Assay

The method for proliferation assay was described previously [4]. The best fit growth curve and doubling time were calculated using GraphPad Prism 5 (GraphPad Software, Inc., San Diego, CA). Briefly, H2M (1×10^4^), H2P (1×10^4^), HepG2 (2×10^4^), MHCC97L (1×10^4^) or MIHA (2×10^4^) cells were plated onto 6-well plates containing complete medium on Day 0. Either vehicle control (DMSO) or IPA-3 (5 or 10 µM) was added into the medium on Day 1. Media with or without IPA-3 were refreshed on Day 3 and 5. In triplicates, cells were trypsinzed and counted using cell counter on Day 3, 4, 6 and 7 for the construction of growth curve.

### Colony Formation Assay

The assay was performed as described previously [14]. Briefly, cells were seeded at 200 cells per well in 6-well plates containing complete DMEM on Day 0. DMSO or IPA-3 (5 or 10 µM) was administrated to the media, which were refreshed two times a week. On Day 14, colonies were fixed with 3.7% formaldehyde for 15 minutes and stained with 1% crystal violet before quantification.

### BrdU Incorporation Assay

The assay was performed as described previously [15]. Cell proliferation was quantified by the measuring the BrdU incorporation during DNA synthesis with the Cell Proliferation ELISA, BrdU Colorimetric kit (Roche Diagnostics). The assay was performed according to the manufacturer’s manual. In brief, equal number of H2M, H2P, MIHA, HepG2 or MHCC97L cells was plated in 96-well plates and serum-starved overnight. Serum starvation induced cell-cycle synchronization so that most of the cells stay in the G1/S transition just before the S phase entry. Cells were then treated with either DMSO or various concentration of IPA-3 (10 and 20 µM) for 15 minutes, followed by FBS replenishment and BrdU labeling for 2, 4 or 8 hours. The BrdU labeling signal was quantified by measuring the relative absorbance (Abs_370 nm_-Abs_492 nm_). Each assay was done in triplicate. The experiments were performed at least three times independently.

### Annexin V-7ADD Staining Assay

Detection of apoptotic cell death was performed using the PE Annexin V Apoptosis Detection Kit I (BD Pharmingen). In triplicate, H2M cells were plated in 60 mm dish, serum-starved overnight and then treated with DMSO or IPA-3 (10 or 20 µM) in medium containing serum for 24 hours. Floating cells were washed away, while the attached cells were trypsinized, rinsed and resuspended in binding buffer. After staining with annexin V-PE and 7-AAD, samples were immediately analyzed by flow cytometry. Excitation: 488 nm (annexin V-PE and 7-AAD). Emission: 578 nm (annexin V-PE), 675 nm (7-AAD). Cells were counted per sample and the data were analyzed with WinMDI (Version 2.8, Joe Trotter).

### Confocal Microscopy

After drug treatment, H2M or H2P cells were fixed in 4% paraformaldehyde for 15 minutes, washed, and permeabilized with 0.2% Triton X-100 in PBS for 15 minutes [4], [15]. Slides were stained with TRITC-phalloidin (Invitrogen) for 10 minutes and immunofluorescence imaging was captured in a Carl Zeiss LSM700 laser confocal scanning microscope.

### Transwell Migration Assay

Transwell migration assay was performed as described previously [4]. Briefly, H2M (1×10^5^) cells were plated in serum-free DMEM at the upper compartment and serum-complemented medium was added to the lower compartment of the Transwell chamber (Corning). In the presence or absence of IPA-3 (10 µM), the serum-starved cells were allowed to migrate for 24 hours. Cells were then fixed with 3.7% formaldehyde, stained with 1% crystal violet and counted under a microscopic field at the magnification of 40X. Three different fields were randomly chosen for each insert.

### Quantitative Reverse Transcription-PCR (qRT-PCR)

Serum-starved H2M cells were treated with or without IPA-3 pretreatment (10 or 20 µM, 15 minutes) followed by TNF-α (10 or 20 ng/ml, 24 hours), FBS replenishment and overnight culture. qRT-PCR was performed as described previously [4]. Briefly, total RNA was extracted using Trizol (Invitrogen) according to the manufacturer’s protocol. Total RNA was reverse transcribed into first-strand cDNA using PrimeScript RT reagent Kit (Takara, Japan). Real-time qPCR was performed using SYBR® Green Real Time system (Takara, Japan) in a My IQTM2 Real-Time PCR Detection System (Bio-Rad). β-actin was used as an internal control and allowed normalization of the samples. DNA sequences of the PCR primers are listed in Table S1. qRT-PCR was performed in triplicates and repeated three times.

### Western Blotting Analysis

Protein extraction and Western blotting were conducted as described previously [4], [15]. Immunoblotting was done for PAK1, P-PAK1 (T423), PARP1, Paxillin, P-Paxillin (S178), SAPK/JNK, P-SAPK/JNK (T183/Y185), cleaved caspase 3 (Cell Signaling Technology), NF-κB (Santa Cruz Biotechnology) and β-actin (Sigma-Aldrich) followed by corresponding horseradish-peroxidase (HRP) conjugated secondary antibodies (GE Healthcare). Signals of targeted proteins were detected by Enhanced Chemiluminsence (GE Healthcare). Intensities of protein bands were analyzed using Adobe Photoshop CS4.

### Nude Mouse Xenograft

MHCC97L cells (1×10^6^) were injected subcutaneously into the right flank of 4-week-old male nude mouse. Tumor size was calculated as described previously [4]. Mice with tumors of a mean size of 100 mm^3^ were grouped into treatment cohorts. A total of 15 mice were used and divided into three groups (5 mice per group): Control (DMSO), IPA-3 (2 mg/kg) and IPA-3 (4 mg/kg). IPA-3 was formulated in DMSO and administrated three times weekly (TIW) (2 mg/kg or 4 mg/kg) by intraperitoneal injection (i.p.) during the study and the tumor size was recorded twice a week. Animal studies had been specifically approved by Animal (Control of Experiments) Ordinance Chapter 340, the Department of Health, Hong Kong Special Administrative Region (Ref.: (11–786) in DH/HA&P/8/2/3 Pt. 33).

### Statistical Analysis

Experiments were done in triplicates and data were presented as mean ± SD. Student’s *t*-test was used for statistical analysis, and data from more than two groups were analyzed by one-way analysis of variance (ANOVA) in GraphPad Prism 6 followed by Dunnett’s test. Results were considered significant when *P*<0.05.

## Results

### IPA-3 Inhibits the Activity of PAK1

**Figure 1:**
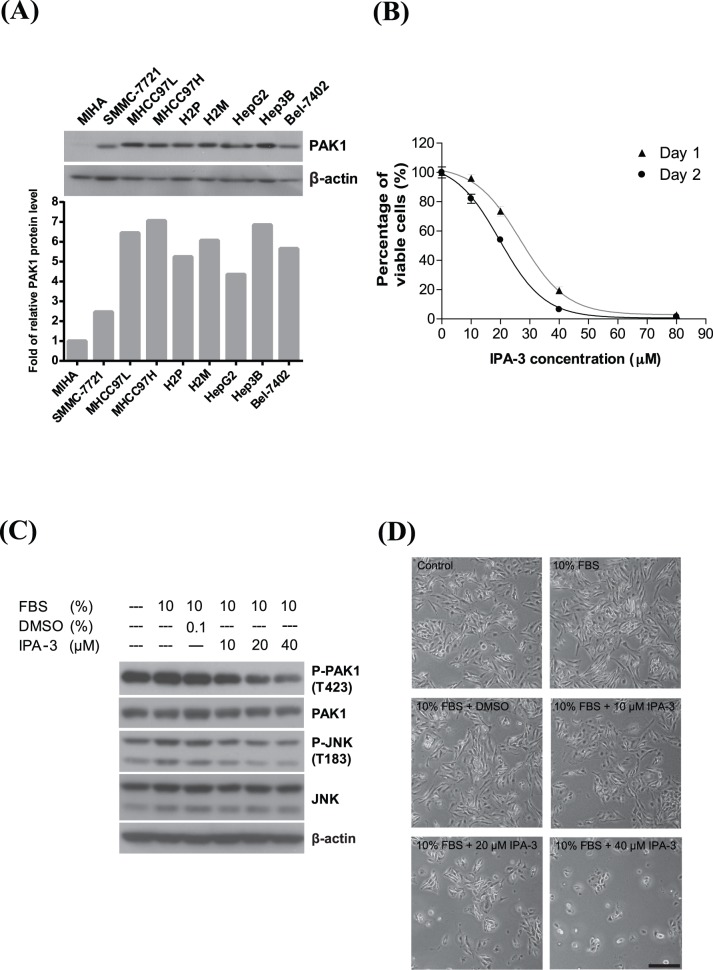
Inhibitory effect of IPA-3 on PAK1. (**A**) Relative protein levels of PAK1 in various cell lines. The signal intensities of the bands were quantified and normalized by taking that level of MIHA as 1. (**B**) MTT assay on Day 1 and 2. H2M cells (4×10^3^) were seeded onto 96-well plates and treated with different dosages of IPA-3. The cells were harvested after incubation and the MTT assay was performed as mentioned in Material and Method. The graph showed the percentage of viable cells plotted against the dosage of IPA-3 (**C**) Western blotting analysis of the P-PAK1, total PAK1, P-JNK and total JNK. Serum-starved H2M cells were treated with either DMSO or IPA-3 at the indicated concentration for 15 minutes and followed by FBS replenishment and overnight culture. (**D**) H2M cells were serum-starved and treated with IPA-3 (10, 20 or 40 µM) in complete medium and allowed to grow overnight. The cell morphology images were captured at a magnification of 40X. Scale bar, 0.4 mm.

HCC cells have been shown to have a high endogenous PAK1 level, particularly in the highly metastatic HCC cell lines [4], such as H2M, but not in the non-tumorigenic, immortalized liver cells, MIHA (Fig. 1A). To investigate the effect of IPA-3 on the growth of H2M cells, MTT assay was performed. MTT assay demonstrated that IPA-3 suppressed the proliferation of H2M cells in time- and dose-dependent manners (Fig. 1B). The half maximal inhibitory concentration (IC_50_) of IPA-3 in H2M cells was about 28 µM and 21 µM on day 1 and 2, respectively. To confirm the inhibitory effect of IPA-3 on PAK1 activity, H2M cells were serum-starved and then treated with IPA-3 in complete medium overnight. The Western blotting analysis showed that IPA-3 dose-dependently reduced the phosphorylation level of PAK1 (Fig. 1C). Consistently, the results showed IPA-3 causes a decrease in PAK1 phosphorylation in association with a decrease in H2M cell viability. In addition, phosphorylation of the downstream substrate of PAK1, c-Jun N-terminal kinase (JNK), was also reduced, further supporting the reduction of PAK1 kinase activity by IPA-3 (Fig. 1C). Light microscopic examination of the IPA-3-treated cells revealed pronounced morphologic changes. Fig. 1D showed the morphologic changes in H2M cells after being treated with IPA-3. In high dosages of IPA-3(20 and 40 µM), a significant population of H2M cells became round-up and detached from the dish.

### IPA-3 Suppresses Proliferation of HCC Cells

**Figure 2:**
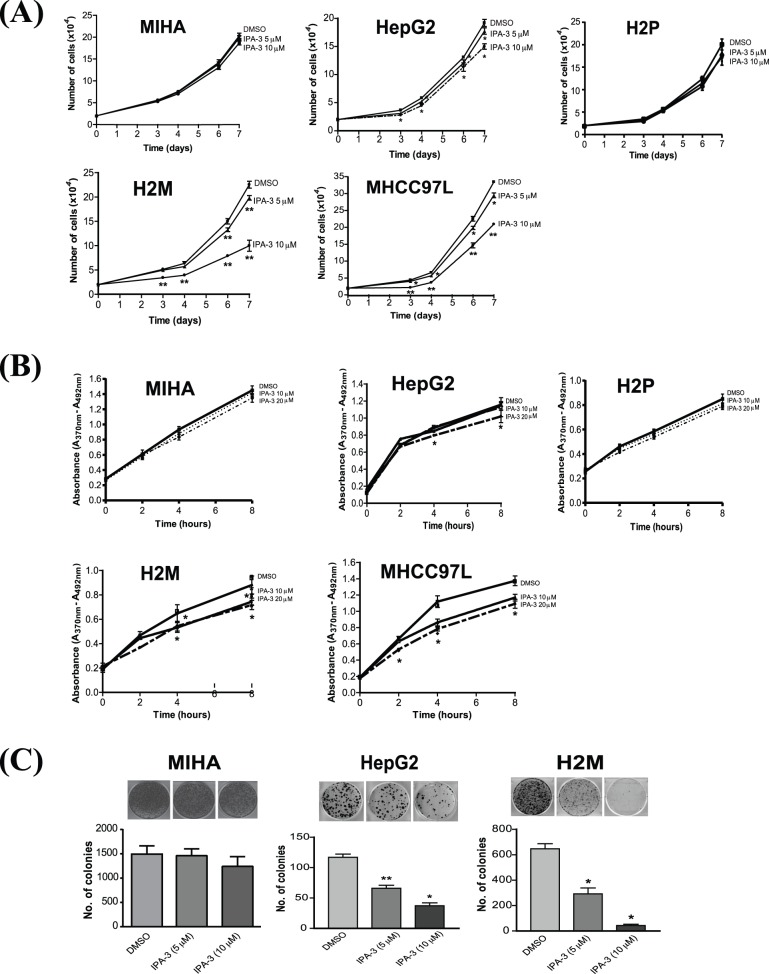
IPA-3 suppressed cell proliferation. (A) The effect of IPA-3 on the cell proliferation rates of MIHA (upper, left panel), HepG2 (upper, middle panel) H2P (upper, right panel), H2M (lower, left panel) and MHCC97L (lower, right panel) cells. Statistical analysis was performed by comparing with the value of DMSO control. *P<0.05, **P<0.01 (ANOVA). (**B**) BrdU labeling assays of MIHA (upper, left panel), HepG2 (upper, middle panel), H2P (upper, right panel), H2M (lower, left panel) and MHCC97L (lower, right panel) cells. *P<0.05 (ANOVA) compared with the DMSO control. Error bars, mean ± SD of triplicate samples. (**C**) Representative plates of colony formation assay of MIHA (left panel), HepG2 (middle panel) and H2M cells (right panel). Bar chart of colony formation assay (lower panel). *P<0.001, **P<0.01 (ANOVA) compared with the DMSO control. Error bars, mean ± SD of triplicate samples.

To further evaluate the effect of IPA-3 on HCC cell proliferation, two primary HCC cell lines, HepG2 and H2P, two metastasis HCC cell lines,. H2M and MHCC97L, and the non-tumorigenic, immortalized liver cell line, MIHA, were separately treated with different dosages of IPA-3. In the cell proliferation assay, treatment of IPA-3 significantly reduced the number of metastatic HCC cells (H2M and MHCC97L) and a lesser extent for the primary HCC cells (HepG2 and H2P), scored in a dose-dependent manner (Fig. 2A). In contrast, MIHA had the highest chemoresistance to IPA-3, suggesting that IPA-3 could inhibit hepatoma cell proliferation with a little effect on normal hepatocytes. To confirm the effect of IPA-3, total cell lysates of H2M cells were collected on Day 7 and tested for PAK1 inhibition by Western blotting analysis. The result showed that IPA-3 treatment markedly inhibited the activating phosphorylation of PAK1, suggesting that IPA-3 inhibits cell proliferation by reducing the PAK1 activity (Supplementary Fig. S1). Consistently, concomitant reduction of the phosphorylation of JNK, the downstream targets of PAK1, was also observed (Fig. S1). To elucidate whether the anti-proliferative mechanism of IPA-3 on HepG2, H2P, H2M and MHCC97L cells was due to a decrease in cell cycle entry, BrdU labeling assay was performed. In order to achieve a prominent effect of IPA-3, 10 µM and a higher dosage (20 µM) were used for the investigation. Our data showed that IPA-3 treatment on the synchronized H2M and MHCC97L cells resulted in a significant reduction in the rate of BrdU incorporation, as compared with the DMSO control in time- and dose-dependent manners (Fig. 2B). However, IPA-3 only had a marginal effect on the BrdU incorporation rate of H2P and HepG2 cells, implicating that IPA-3 is highly specific for the metastatic HCC cell lines. To further confirm the effect of IPA-3 on the suppression of HCC cell growth, colony formation assay was performed using non-tumorigenic (MIHA), primary (HepG2) and metastatic (H2M) HCC cells. The result showed that IPA-3 significantly inhibited the growth of HepG2 and H2M cells, but merely had a very marginal effect on MIHA cells (Fig. 2C). Noteworthy, MIHA cells, which have a very low endogenous level of PAK1, showed no difference in cell proliferation upon IPA-3 treatment. Taken together, these results indicated that IPA-3 treatment suppressed HCC cell proliferation in a descending order of preference, metastatic HCC >primary HCC>non-transformed hepatocytes, and in a PAK1-dependent manner.

### IPA-3 Induces Apoptosis of HCC Cells

**Figure 3:**
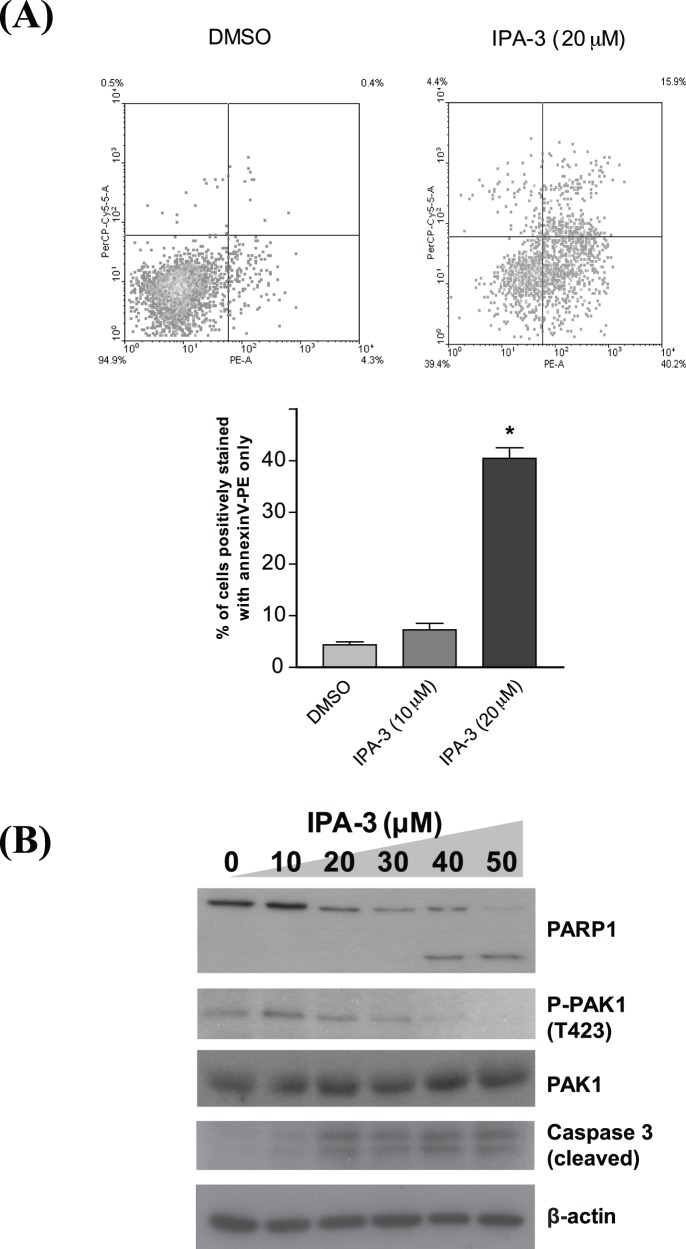
The induction of apoptosis by IPA-3. (**A**) Annexin V-7ADD staining assay. The fluorescence signals of annexin V-PE and 7-AAD were detected with PE-A and Per-Cy5-5-A channels, respectively (upper panel). The percentage of H2M cells stained with annexin V-PE only under different treatments was shown in a bar chart (lower panel). *P<0.001 (ANOVA) compared with the DMSO control. (**B**) Serum-starved H2M cells were treated with increasing dosages of IPA-3 together with serum replenishment for 24 hours. Western blotting analysis of PARP1, P-PAK1 (T423), total PAK1 and cleaved caspase 3 was performed.

To investigate whether IPA-3 affects apoptosis in HCC cells, an annexin V-7ADD staining assay was performed. Since 10 µM IPA-3 inhibits proliferation rate and a higher concentration i.e. 20 µM causes a significant result in the BrdU incorporation assay in H2M cells, 10 µM and 20 µM were used to investigate the prominent effect of IPA-3. The result showed that incubation of H2M cells with 20 µM IPA-3 led to a higher percentage of cells displaying a positive signal of annexin V staining, as compared with the DMSO control, suggesting that cells underwent apoptosis under the treatment with IPA-3 (Fig. 3A). In addition, the potential pro-apoptotic activity of IPA-3 was further examined by the cleavage of PARP1 and caspase 3. Treatment of IPA-3 in H2M cells resulted in an attenuated level of PARP at 20 µM and the cleavage form of PARP1 could be detected at 40 µM (Fig. 3B). This was accompanied with a dose-dependent reduction of PAK1 phosphorylation levels of PAK1 (Fig. 3B). In addition, the level of cleaved caspase 3 increased in a dose-dependent manner. Taken together, these results suggested that IPA-3 induces apoptosis through PAK1 inhibition at a high concentration.

### IPA-3 Suppresses HCC Cell Migration

**Figure 4:**
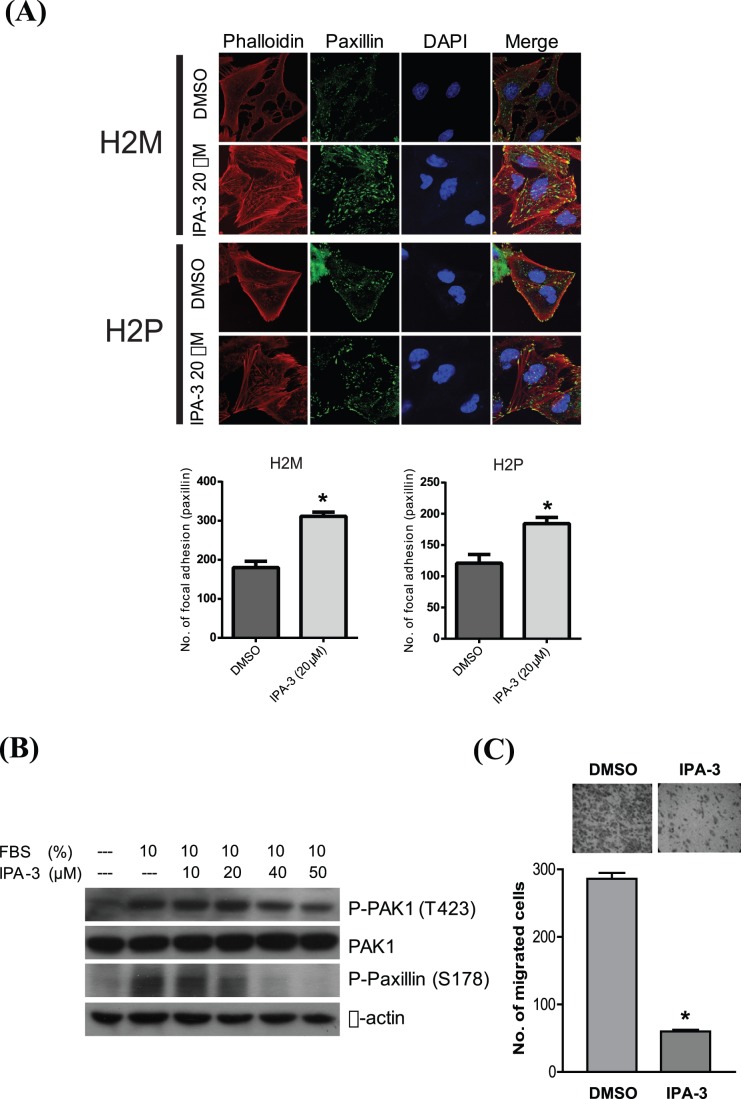
The suppressive effect of IPA-3 on migration of H2M cells. (**A**) Paxillin protein expression was detected by immunofluorescence analysis under IPA-3 treatment. H2M and H2P (upper panel) cells were serum-starved overnight and treated with either DMSO control or IPA-3 (20 µM) for 15 minutes, followed by FBS replenishment for 10 minutes. Immunofluoresence signals of phalloidin (Red), paxillin (Green) and DAPI (Blue) represent stress fiber, focal adhesion and nucleus, respectively (magnification 40X). The number of focal adhesion (paxillin) were counted in H2M (lower, left panel) and H2P (lower, right panel), and represented in the bar chart. Error bars, mean ± SD of triplicate samples. *P<0.01 (*t*-test) compared with DMSO control. (**B**) Western blotting analysis on the phosphorylation levels of PAK1 and paxillin. Serum-starved cells were treated with various concentrations of IPA-3 as indicated for 15 minutes, followed by FBS replenishment for 10 minutes. (**C**) Representative images of Transwell migration assay of H2M cells. Cells were treated with either DMSO or 10 µM IPA-3, and were allowed to migrate for 24 hours. Images show the cells having migrated to the lower chamber (upper panel). The number of migrated cells were counted and represented in the bar chart (lower panel). Error bars, mean ± SD of triplicate samples. *P<0.01 (*t*-test) compared with DMSO control.

PAK1 has a prominent role in cell migration, particularly in the regulation of stress fibers formation and focal adhesions turnover. Thus, immunofluorescence staining was performed to investigate whether IPA-3 can influence these processes. IPA-3 was found to enhance the formation of stress fibers and focal adhesions in both H2M and H2P cells, which were visualized by the phalloidin and paxillin immuno-positive signals of stress fibers and focal adhesions, whereas the DMSO control showed a weak and scattered staining. In addition, the results showed that treatment of IPA-3 significantly enhanced the number of focal adhesion complexes in H2M and H2P cells as revealed by paxillin staining (Fig. 4A). Phosphorylation of paxillin at serine-178 (S178) is important for PAK1-mediated cell migration, which has been showed to be phosphorylated by JNK [4], [16]. In H2M cells, phosphorylation of paxillin at S178 was significantly reduced along with an overnight treatment of IPA-3 (Fig. 4B). Thus, IPA-3 inhibits the signaling of the PAK1/JNK/paxillin pathway. Moreover, a Transwell migration assay was performed to study the effect of IPA-3 on the *in vitro* migration ability of H2M cells. We found that IPA-3 significantly suppressed the migration of H2M cells as the number of migrated cells was remarkably reduced by 79%, as compared with the DMSO control (Fig. 4C). Taken together, these data illustrated that IPA-3 significantly reduces the PAK1-dependent cell mobility of HCC cells.

### IPA-3 Suppresses NF-κB Nuclear Translocation

**Figure 5:**
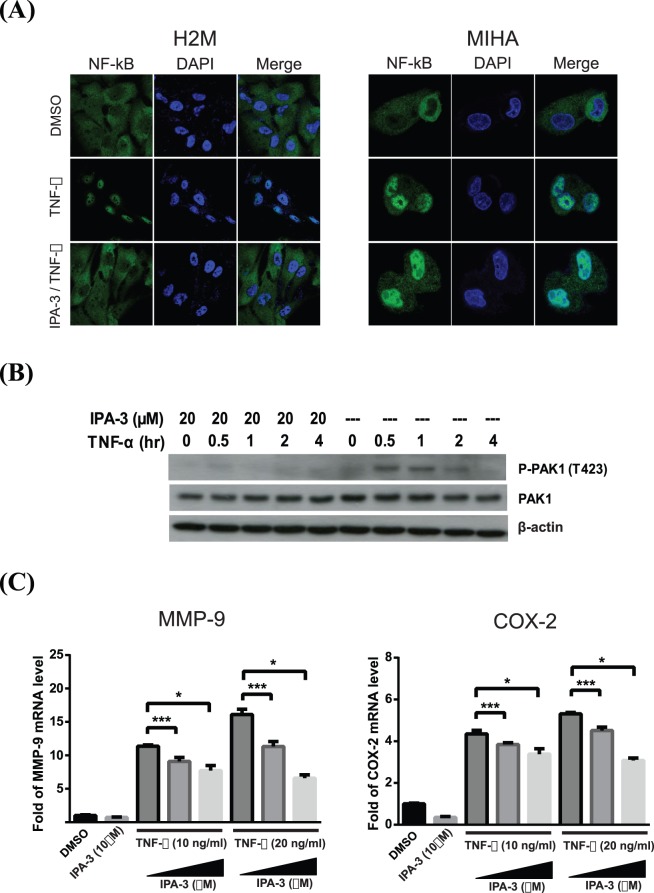
The inhibitory effect of IPA-3 on NF-κB nuclear translocation. (**A**) Effect of IPA-3 on subcellular localization of NF-κB was evaluated by immunofluorescence staining. After overnight serum starvation, H2M (left panel) and MIHA (right panel) cells were treated with either DMSO or IPA-3 (20 µM, 15 minutes) followed by an addition of TNF-α (20 ng/ml, 15 minutes). NF-κB was detected with a specific antibody (Green) and nucleus was stained with DAPI (Blue). (**B**) Western blotting analysis of P-PAK1 (T423) and total PAK1 were detected in the H2M cells stimulated by TNF-α (20 ng/ml) with or without IPA-3 pretreatment (20 µM, 15 minutes). TNF-α was included in the culture medium for 0, 0.5, 1, 2 or 4 hours. (**C**) Expression of quantitative real-time PCR was performed to analyze the mRNA level of MMP-9 (left panel) and COX-2 (right panel). Serum-starved H2M cells were treated with or without IPA-3 pretreatment (10 or 20 µM, 15 minutes) followed by TNF-α (10 or 20 ng/ml, 24 hours). Quantitative results of MMP-9 and COX-2 mRNA levels were normalized to β-actin. The values represented the mean ± SD of three independent experiments. *P<0.001 (ANOVA), ***P<0.05 (ANOVA) compared with the TNF-α control.

Previous reports demonstrated that PAK1 stimulates the activity and subcellular translocation of nuclear factor light-chain enhancer of activated B cells (NF-κB), and promotes cell survival [17], [18]. Thus, we examined whether IPA-3 is able to inhibit the activity of NF-κB. H2M with a high endogenous expression level of PAK1 and immortalized hepatocytes, MIHA cells were serum-starved and then separately treated with IPA-3 followed by tumor necrosis factor-alpha (TNF-α). As showed in Fig. 5A, NF-κB positive staining was predominantly detected in the cytoplasm of the DMSO control, whereas NF-κB staining was accumulated in the nucleus after TNF-α induction. Interestingly, H2M cells pretreated with IPA-3 resulted in a cytoplasmic staining of NF-κB, indicating that IPA-3 suppressed the TNF-α-induced nuclear targeting of NF-κB. Unlike H2M cells, IPA-3 did not suppress TNF-α-induced NF-κB activation in MIHA cells, which lack the endogenous PAK1. This result suggested that the inhibition of NF-κB activation by IPA-3 is PAK1-dependent. To further investigate whether PAK1 inactivation is involved in the IPA-3-induced suppression of NF-κB translocation, phosphorylation of PAK1 was determined (Fig. 5B). Consistent with an earlier study which demonstrated that PAK1 was promptly activated by TNF-α in various cell lines [19], the phospho-PAK1 level was elevated in cells stimulated with TNF-α (20 ng/ml). The phosphorylation level was highest at 0.5-hour and then gradually reduced afterwards. On the other hand, the phosphorylation of PAK1 was completely suppressed in IPA-3 pretreated cells. This result suggested that IPA-3 is able to abolish the PAK1 activation induced by TNF-α, which correlated well with the IPA-3 inhibition on TNF-α-induced NF-κB translocation. The induction of metalloproteinase (MMP)-9 by TNF-α was shown to be mediated by PAK1 [19]. To elucidate the effect of IPA-3 on the TNF-α-induced activity of MMP-9 and COX-2, which are downstream targets of NF-κB [20], [21], we performed qRT-PCR to quantify the mRNA production of MMP-9 and COX-2. After normalization with β-actin, H2M cells responded to TNF-α with an increasing mRNA production of MMP-9 and COX-2 (Fig. 5C). However, the results showed that treatment of IPA-3 significantly suppressed the expression of MMP-9 and COX-2 transcripts in a dose dependent manner.

### IPA-3 Suppresses Tumorigenesis in Nude Mouse Xenograft Model

**Figure 6:**
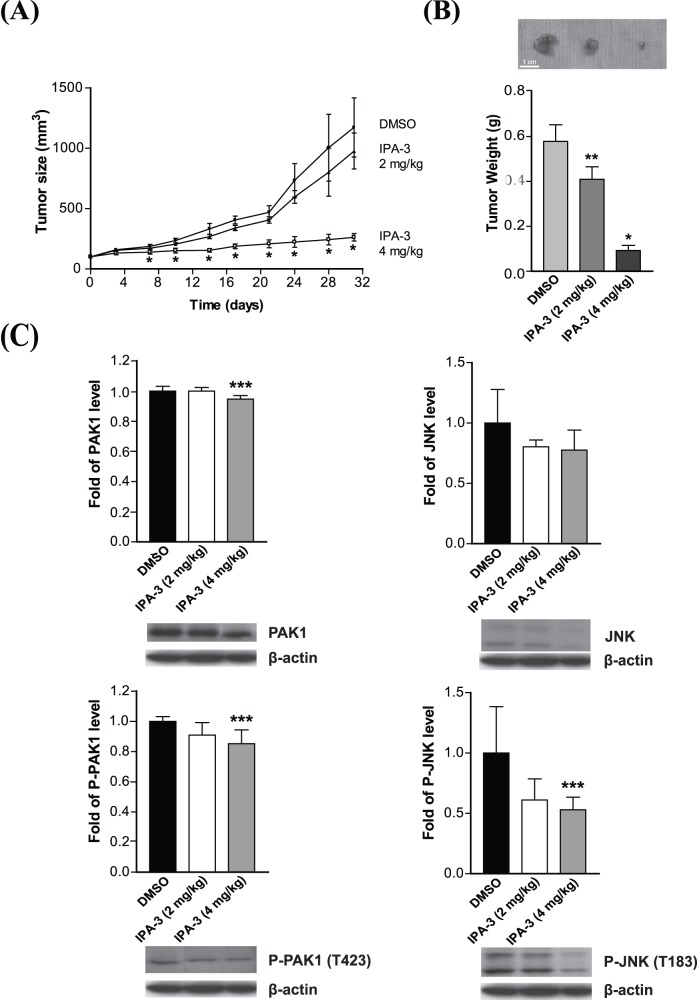
The suppressive effect of IPA-3 in nude mouse xenograft model. (**A**) MHCC97L cells were used for the xenograft model. Mice were treated three times weekly either with DMSO or IPA-3 (2 mg/kg or 4 mg/kg, i.p.). *P<0.001 (ANOVA) compared with the DMSO control group. (**B**) Tumor weights were measured at the end of study. *P<0.001, **P<0.01, (ANOVA) compared with the DMSO control group. (**C**) Representative results of Western blotting analysis. P-PAK1 (T423), total PAK1, P-JNK and total JNK were detected. ***P<0.05 (ANOVA) compared with the DMSO control. Error bars, mean ± SD of 5 animals per group.

The breadth of IPA-3 antitumor activity *in vivo* was evaluated using a nude mouse xenograft model. Since the H2M cell line was unable to develop solid tumors in nude mice, another human HCC line MHCC97L with similar PAK1 level was used (Fig. 1A). Following tumor establishment (100 mm^3^), five mice per groups were treated with either DMSO or various doses of IPA-3 (2 mg/kg and 4 mg/kg) TIW by i.p. injection. While the treatment was well tolerated as proved by no significant weight loss, IPA-3 significantly suppressed tumor growth (Fig. 6A) and resulted in lower tumor weights (Fig. 6B). In addition, Western blotting analysis showed that IPA-3 reduced the phosphorylation of PAK1 and its downstream target JNK (Fig. 6C). Taken together, these results indicated that IPA-3 can suppress tumorigenesis *in vivo* by reducing the PAK1 activity.

## Discussion

PAK1 is involved in a complex signaling transduction network which is linked to various cellular processes, including cytoskeleton modeling, cell motility, survival and proliferation, and cell cycle progression [5]. Deregulated or hyper-activated PAK1 is commonly associated with HCC [19]. Thus, PAK1 has become a potential therapeutic target in controlling tumorigenesis and metastasis of HCC [22]. IPA-3 has been identified to be an allosteric small molecule inhibitor of PAK1 [8]. With little or less reports of IPA-3 in the treatment of human HCC, we employed various *in vitro* and *in vivo* experiments to investigate the anti-tumorigenic ability of IPA-3 treatment on human HCC cell lines.

In this study, we demonstrated that IPA-3 could inhibit the proliferation rate of HepG2, H2P, H2M, and MHCC97L cells in dose- and time-dependent manners. Moreover, IPA-3 significantly inhibited the colony-formation ability of both H2M and HepG2 cells, indicating its suppressive effect on HCC cell proliferation. Also, the results from the BrdU labeling assay indicated that IPA-3 significantly inhibited the H2P, H2M, HepG2 and MHCC97L cell growth in dose- and time-dependent manners. Notably, IPA-3 has no effect on the MIHA cells in all the cell proliferation assays, indicating that non-transformed hepatocytes or cells with a low endogenous PAK1 level are not sensitive to IPA-3. Furthermore, the nude mice xenograft assay demonstrated that IPA-3 can significantly suppress the HCC tumor growth *in vivo.*

Given that PAK1 has a pivotal role in controlling cell proliferation, the suppression on cell proliferation by IPA-3 could be a result of apoptosis induction. We demonstrated that administration of IPA-3 exhibited a pro-apoptotic activity of H2M cells by using annexin V-7ADD staining assay followed by flow cytometry analysis. This result showed that IPA-3 could significantly induce the apoptosis of H2M cells. Moreover, IPA-3 treatment of H2M cells could lead to an up-regulation of cleaved caspase 3 and down-regulation of cleaved PARP1, which is an apoptotic marker, as well as phosphorylated PAK1. Although the mechanism by which IPA-3 induces the apoptosis of HCC cells is not completely known, PAK1 has been shown to phosphorylate the anti-apoptotic protein, BAD, to suppress the apoptosis of cancer cells [23]. Taken together, these results suggested that the inhibitory effect of IPA-3 on HCC cells may be attributed to the suppression of PAK1-mediated inhibition of apoptosis.

Previous studies reported that PAK1 enhance HCC cell migration by downregulating the formation of stress fiber and focal adhesion complex [4]. In the present study, firstly, we demonstrated the inhibitory effect of IPA-3 on FBS- and TNF-α-induced phosphorylation of PAK1 in HCC cell lines, which was consistent with the earlier findings that IPA-3 impeded both basal and PDGF-stimulated PAK1 activation in fibroblasts [8]. Secondly, we showed that IPA-3 was capable to stabilize these formations, most likely through the inhibition of PAK1 activity by using immunofluorescence staining. Lastly, we further confirmed that IPA-3 treatment significantly suppressed the motility of H2M cells with a high endogenous level of PAK1 using the Transwell migration assay. Taken together, these results provided the first evidence that IPA-3 can inhibit HCC cell migration through the inactivation of PAK1.

To elucidate the molecular mechanism by which IPA-3 inhibited the HCC cell survival, we examined the effect of IPA-3 in the regulation of NF-κB activity. Constitutive activation of NF-κB transcription factors is suggested to be associated with cancer malignancy by conferring an anti-apoptotic effect to cells [24]. Hence, the inhibitory effect on TNF-α-induced NF-κB activity by IPA-3 might suggest a potential use for controlling HCC tumorigenesis. Our data demonstrated that treatment of IPA-3 not only abolished the nuclear translocation of NF-κB, but also remarkably inhibited the activity of the NF-κB transcriptional targets, MMP-9 and COX-2, which were also induced by TNF-α. This result correlated well with the earlier findings on human dermal fibroblasts with an overexpression of a dominant inactive (K299R) mutant of PAK1 that TNF-α-induced expression and activation of MMP-9 were highly dependent on PAK1 activity [19]. Moreover, the results of this study showed that NF-κB activation contributed to TNF-α-induced COX-2 induction in H2M cells and IPA-3 inhibited TNF-α-induced COX-2 gene expression. This result is consistent with the previous findings that the reduction in PAK1 with siRNA decreased COX-2 expression in papilloma cells and reduced nuclear localization of NF-κB [25]. In addition, we assessed the anti-tumorigenic ability of IPA-3 by a nude mouse xenograft assay. IPA-3 showed a significantly reduction in tumor growth from the MHCC97L cells and the effect of IPA-3 on PAK1 activity was demonstrated by the reduction of the phosphorylation levels of PAK1 and JNK. These data further support the reduction of *in vitro* PAK1 kinase activity, suggesting that IPA-3 regulates HCC tumorigenesis by inhibiting the PAK1/JNK axis.

Apart from IPA-3, many inhibitors have been shown to suppress PAK1 activity. However, those targeting the evolutionary conserved ATP-binding pocket of kinases may not be specific and cause drug toxicity [26], [27]. By allosteric inhibition, the non-ATP competitive inhibitor, IPA-3, achieves a remarkably high selectivity that only exhibited off-target effects (>50% inhibition) on 9 out of 214 kinases tested [8]. In our study, IPA-3 treatment suppresses both *in vitro* and *in vivo* HCC cell growth, at least in part, via the inhibition of PAK1. These results clearly explain that IPA-3 is able to inhibit PAK1 activity not only prior to autophosphorylation. Furthermore, PAK1 is rapidly cycling between the active and inactive states [28] and this increases the opportunity for IPA-3 to interact with the inactivated PAK1 in living cells. Upon binding of IPA-3 to its regulatory domain, PAK1 is believed to undergo a conformational change where T423 is exposed [8], [9]. Moreover, the catalytically inactive PAK1 cannot be reactivated via phosphorylation by exogenous kinases [8].

In summary, our data demonstrated that IPA-3 can suppress HCC cell proliferation and motility, enhance cell death as well as restrain liver tumor development, progression and tumorigenicity by modulating the regulators involved in proliferation, migration and apoptosis. The current study provides supportive evidences for the potential application of IPA-3 in HCC treatment and the specificity of IPA-3 in targeting PAK1, suggesting that IPA-3 is a potential therapeutic target for the treatment of HCC and may be more effective on the advanced cancers. One of the possible explanations for that is the high endogenous PAK1 activity in the metastatic HCC cells.

## Supporting Information

Figure S1 **The effect of IPA-3 on PAK1.** Western blotting analysis of P-PAK1 (T423), total-PAK1, P-JNK, total-JNK and P-paxillin in the H2M cell lysates extracted after 6 days of IPA-3 treatment.
(EPS)

Table S1 **Primers used for quantitative RT-PCR.**
(EPS)
